# Carbon NanoFiber-Integrated VN@CNS Multilevel Architectures for High-Performance Zinc-Ion Batteries

**DOI:** 10.3390/mi16111265

**Published:** 2025-11-10

**Authors:** Yun Cheng, Taoyun Zhou, Jianbo Wang, Yiwen Wang, Xinyu Li

**Affiliations:** 1 School of Information, Hunan University of Humanities, Science and Technology, Loudi 417000, China; 2 Faculty of Physics and Electronic Information Engineering, Guilin University of Technology, Guilin 541004, China

**Keywords:** aqueous zinc-ion batteries (AZIBs), vanadium nitride (VN), carbon coating, hierarchical tandem structure, electrochemical performance

## Abstract

Aqueous zinc-ion batteries (AZIBs) have attracted considerable attention due to their intrinsic safety, low cost, and environmental friendliness. However, drastic volume expansion, sluggish reaction kinetics, and the insufficient structural stability of electrode materials still remain key challenges. In this work, a cascade structure-guided electron transport strategy was used to construct a vanadium nitride@carbon nanosheet/carbon nanofiber (VN@CNS/CNF) composite as a high-performance cathode for AZIBs. In this rationally engineered architecture, carbon-coated VN nanoparticles are uniformly anchored on a conductive carbon nanofiber network, forming a multidimensional interconnected structure that enables fast electron/ion transport and robust mechanical stability. The carbon shell effectively alleviates volume expansion and prevents VN nanoparticle agglomeration, while the continuous carbon fiber backbone reduces charge transfer resistance and enhances reaction kinetics. Benefiting from this synergistic structural design, the VN@CNS/CNF electrode delivers a high specific capacity of 564 mAh g^−1^ at 0.1 A g^−1^, maintains 99% capacity retention after 50 cycles, and retains 280 mAh g^−1^ even at 8 A g^−1^ after prolonged cycling. This study provides a new structural engineering strategy for vanadium nitride-based electrodes and provides strategic guidance for the development of fast-charging, durable aqueous zinc-ion batteries.

## 1. Introduction

Aqueous zinc-ion batteries (AZIBs) have emerged as one of the most promising candidates for next-generation energy storage systems, owing to their intrinsic safety, environmental friendliness, and the abundance of zinc metal resources. These batteries offer significant advantages, including low cost and a high theoretical capacity, making them an attractive alternative to traditional lithium-ion batteries for large-scale energy storage applications [1,2,3,4,5]. Among the key components of AZIBs, the selection of an appropriate cathode material plays a crucial role in determining the overall electrochemical performance of the battery [6]. In particular, much of the research to date has concentrated on manganese- and vanadium-based compounds, as well as Prussian blue analogs, with vanadium-based materials being particularly well studied due to their superior properties [7,8,9,10].

In recent years, vanadium nitride (VN) has gained significant attention as a promising cathode material for AZIBs, thanks to its excellent electrical conductivity, high theoretical capacity, and robust stability in aqueous electrolytes [11,12,13,14,15]. Despite these advantages, VN cathode materials face several challenges in practical applications, including substantial volume expansion, insufficient structural stability, and sluggish reaction kinetics. These issues severely hinder the electrochemical performance and long-term cycling stability of AZIBs [16,17].

To address these challenges, various strategies have been proposed, including defect engineering, nanostructure design, conductive matrix composites, heterostructure construction, and heteroatom doping [18,19,20,21,22]. Recent advancements in nanostructured materials have shown that reducing the particle size of VN, such as synthesizing VN nanosheets, can significantly improve surface redox pseudocapacitance, which exposes more active sites and enhances energy storage performance. For example, Wei et al. demonstrated that reducing the particle size of VN from 50 nm to 10 nm during the synthesis of VN nanosheets led to a significant increase in specific capacity [23]. However, while nanosized VN materials improve the surface pseudocapacitance effect, their high surface energy makes them prone to agglomeration, and the volume expansion during electrochemical cycling can lead to structural collapse, resulting in capacity decay [24,25].

Recent studies have emphasized the importance of structural optimization to mitigate these issues. Constructing three-dimensional conductive networks through multilevel structural designs has been shown to be an effective approach. For example, the VN/N-CNF composites developed by Sun’s team using electrostatic spinning technology demonstrated a spatially confined domain of active sites, maintaining a capacity of 482 mAh g^−1^ even at a high rate of 50 A g^−1^ [26]. This work highlighted the critical role of interfacial engineering and structural design in enhancing the comprehensive electrochemical performance of AZIBs. Additionally, the integration of carbon nanosheets (CNSs) and carbon nanofibers (CNFs) can help maintain the mechanical stability of the composite material, prevent agglomeration, and provide efficient pathways for ion and electron transport. These findings are in line with recent studies, such as those presented in *Joule* 2020 [27] and *Energy Environ. Sci.* 2025 [28], which underscore the significance of core–shell and multistage composite designs in improving the stability and performance of electrode materials for energy storage devices. In [27], core–shell nanostructures were shown to significantly enhance cycling stability and rate capability by mitigating volume expansion during cycling. Similarly, ref. [28] highlighted the benefits of multistage composites, demonstrating enhanced ion diffusion and improved electron transport through the integration of carbon nanosheets and carbon nanofibers.

Building upon these advancements, this study presents a novel strategy for designing VN-based cathodes by constructing VN@CNS/CNF multistage composites with a unique tandem structure. This composite design incorporates carbon-coated VN nanosheets (VN@CNS) as the core units, which are uniformly anchored to a three-dimensional conductive carbon nanofiber (CNF) skeleton via interfacial bonding. This tandem architecture creates efficient multistage electron and ion transport pathways, reducing interfacial resistance and optimizing the transport of both electrons and ions. Furthermore, the carbon shell’s spatial confinement effect mitigates the agglomeration of VN nanoparticles and limits the volume expansion during cycling, thereby significantly enhancing the structural stability of the electrode. This strategy is consistent with [27,28], where similar core–shell and composite structures have shown remarkable electrochemical stability and high capacity retention, even under extreme cycling conditions.

As a result, the composite material exhibits a remarkable specific capacity of 564 mAh g^−1^ at 0.1 A g^−1^, with exceptional cycling stability, retaining 99% of its capacity after 50 cycles. Notably, the material maintains a capacity of 280 mAh g^−1^ even at an ultrahigh rate of 8 A g^−1^ after 1000 cycles. These findings demonstrate the potential of this multistage composite strategy for the development of high-performance electrode materials for next-generation energy storage devices, aligning with and expanding upon the recent advancements in the field.

## 2. Materials and Methods

All chemicals were of analytical grade and used without further purification. Ammonium metavanadate (NH_4_VO_3_, ≥99.0%), glucose (C_6_H_12_O_6_, ≥99.0%), and zinc trifluoromethanesulfonate [Zn(CF_3_SO_3_)_2_, ≥98.0%] were purchased from Aladdin Chemical Reagent Co., Ltd. (Shanghai, China). Carbon fibers (CFs, diameter ≈ 10 μm) were obtained from Toray Industries, Tokyo, Japan. Deionized water (18.2 MΩ cm, Millipore system, Burlington, MA, USA) was used throughout the experiments.

### 2.1. Synthesis of VN@CNS/CNF

First, 6 mL of dimethyl sulfoxide (DMSO, ≥99.7%) and 12 mL of deionized water were mixed and heated to 50 °C to prepare a homogeneous solution. Then, 1.5 mmol of ammonium metavanadate (NH_4_VO_3_, 99%) was added, and the mixture was stirred until fully dissolved. Next, 4.5 mmol of melamine (C_3_H_6_N_6_, 99.7%) was introduced into the solution, and the mixture was stirred vigorously at 50–70 °C for 1 h. Afterward, 0.1 mol L^−1^ of cetyltrimethylammonium bromide (CTAB) was added as a surfactant to adjust the surface properties of the reaction system. The mixture was then transferred to a Teflon-lined autoclave, and a 1:1 volume ratio mixture of ethanol and toluene was added, occupying 60–80% of the reactor’s volume. The reaction was carried out hydrothermally for 12 h to facilitate the formation of VN nanoparticles.

**Figure 1:**
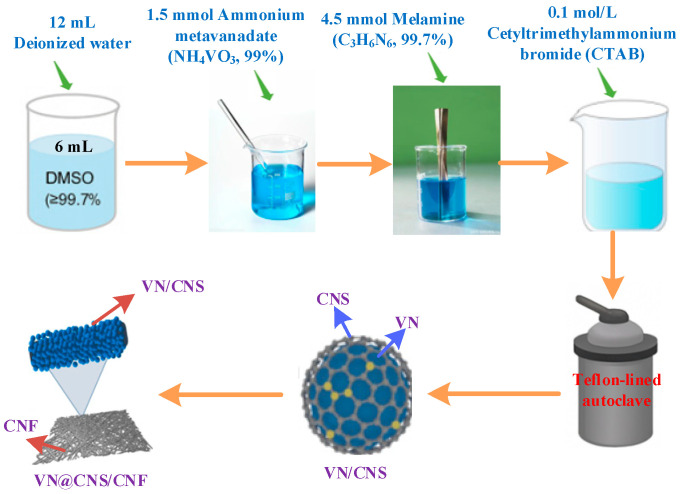
The synthesis procedure of the VN@CNS/CNF composite.

Following the reaction, the product was filtered, washed thoroughly with anhydrous ethanol and deionized water, and then vacuum-dried at 60 °C for 12 h. The dried product was calcined in a flowing argon atmosphere at 600 °C for 1.5 h (heating rate: 5 °C min^−1^). After cooling to room temperature, the final VN@CNS/CNF composite was obtained. The overall synthesis procedure is illustrated in Figure 1.

During this process, the polar functional groups of melamine interacted with the surface of the VN precursor, leading to crosslinking and providing nitrogen and carbon sources. During the hydrothermal reaction, ammonium metavanadate was converted into vanadium nitride (VN), with melamine coating the surface to form the VN@CNS composite precursor. CTAB served as a surfactant, aiding in the uniform wrapping of the precursor. During the subsequent high-temperature heating process, the excess melamine compensated for sublimation effects and provided additional carbon and nitrogen sources. As melamine decomposed, a fibrous structure was formed, which anchored the VN@CNS precursor, eventually leading to the formation of the VN@CNS/CNF multilevel composite structure.

### 2.2. Electrode Preparation and Cell Assembly

The cathode electrodes were prepared as follows: The active material, conductive carbon black (Super-P), and polyvinylidene fluoride (PVDF) binder were accurately weighed at a mass ratio of 7:2:1 and thoroughly ground for 30 min to ensure uniform dispersion. Subsequently, an appropriate amount of N-methyl-2-pyrrolidone (NMP) was added dropwise to form a homogeneous slurry through continuous grinding. The resulting slurry was uniformly coated onto a clean 400-mesh stainless steel current collector using a doctor blade, ensuring even coating thickness, enhanced electrical conductivity and mechanical stability of the electrode film. The coated electrodes were then dried under vacuum at 60 °C for 12 h to completely remove residual solvent and moisture. Finally, the dried films were cut into circular disks with a diameter of 16 mm to obtain the cathode electrodes. For the anode, 16 mm diameter zinc foils were used after carefully polishing to remove any surface oxide layers.

The Zn-ion batteries were assembled in CR2016-type coin cells. The zinc foil served as the anode, a Whatman glass fiber membrane (19 mm in diameter) was used as the separator, and 3 M Zn(CF_3_SO_3_)_2_ aqueous solution served as the electrolyte. The cathode, separator, electrolyte, and zinc foil were sequentially stacked in the cell casing, which was then hermetically sealed using a crimping machine. The assembled cells were allowed to rest for 12 h at room temperature to ensure sufficient activation prior to electrochemical performance measurements.

### 2.3. Experimental Instruments

The morphology and microstructure of the obtained samples were examined using scanning electron microscopy (SEM, JEOL JSM-7900F, JEOL Ltd., Tokyo, Japan) and transmission electron microscopy (TEM, FEI Tecnai G2 F20, FEI Company, Hillsboro, OR, USA). The crystal structures were characterized by X-ray diffraction (XRD, Bruker D8 Advance, Bruker AXS GmbH, Billerica, MA, USA) with Cu Kα radiation (λ = 1.5406 Å) operating at 40 kV and 40 mA. Raman spectra were obtained on a Renishaw inVia Raman spectrometer(Renishaw plc, Gloucestershire, UK) using a 532 nm excitation laser. Thermogravimetric analysis (TGA) was carried out on a Netzsch STA 449C thermal analyzer(Netzsch-Gerätebau GmbH, Selb, Germany) under an air atmosphere with a heating rate of 10 °C min^−1^. Nitrogen adsorption–desorption isotherms were measured at 77 K using a Micromeritics ASAP 2020 surface area analyzer (Micromeritics Instrument Corp., Norcross, GA, USA), and the specific surface area was calculated using the Brunauer–Emmett–Teller (BET) method. The surface elemental composition and chemical states were analyzed by X-ray photoelectron spectroscopy (XPS, Thermo Scientific ESCALAB 250Xi, Thermo Fisher Scientific, Waltham, MA, USA) with Al Kα radiation as the excitation source, and the binding energies were calibrated using the C 1s peak at 284.8 eV.

Electrochemical tests were evaluated using CR2016-type coin cells assembled under ambient conditions. A 3 M aqueous zinc trifluoromethanesulfonate [Zn(CF_3_SO_3_)_2_] solution was used as the electrolyte. The electrochemical performance of the VN@CNS/CNF composite was systematically evaluated using several techniques. Cyclic voltammetry (CV) was first performed to analyze the redox characteristics and reversibility of the electrode material. Following this, galvanostatic charge–discharge (GCD) cycling tests were conducted to assess the long-term cycling stability. The rate performance was evaluated at various current densities to examine the electrochemical response of the VN@CNS/CNF composite. Electrochemical impedance spectroscopy (EIS) was employed to investigate the charge transfer behavior at the electrode/electrolyte interface. Additionally, the galvanostatic intermittent titration technique (GITT) was used to determine the Zn^2+^ diffusion coefficients and gain insights into the ion transport kinetics. All tests were performed within a potential window of 0.2–1.8 V to ensure the reliability and reproducibility of the experimental results.

## 3. Results and Analysis

### 3.1. Characterization of VN@CNS/CNF

#### 3.1.1. SEM and TEM Characterization

**Figure 2:**
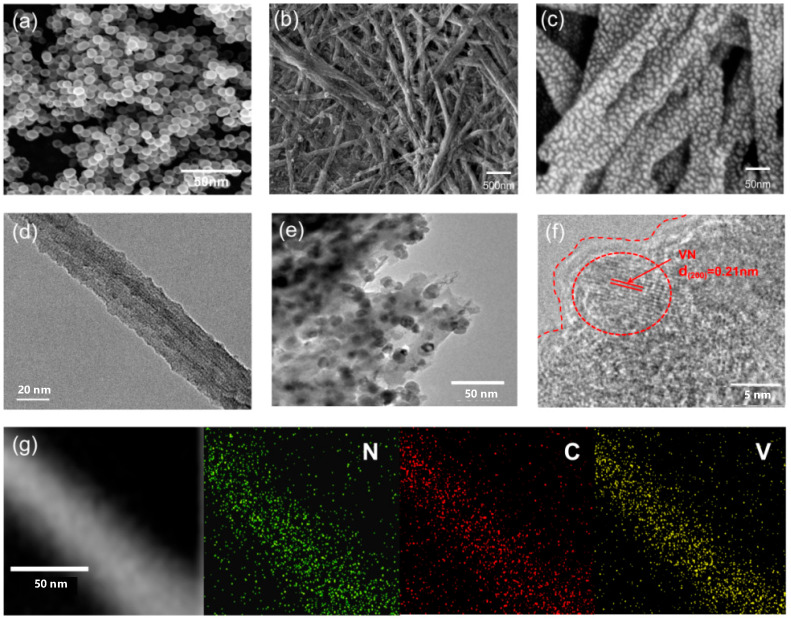
(**a**) SEM image of VN@CNS; (**b**) low-magnification SEM image of VN@CNS/CNF showing interconnected fibrous network; (**c**) high-magnification SEM image revealing uniform distribution of VN nanoparticles on carbon nanofibers; (**d**) TEM image of VN@CNS/CNF; (**e**) high-resolution TEM image displaying carbon-coated VN nanoparticles anchored on CNF surface; (**f**) HRTEM image showing the distinct lattice fringes of VN; (**g**) EDS elemental mapping demonstrating homogeneous distribution of V, N, and C elements within composite.

The microstructural features of the VN@CNS/CNF composites were systematically characterized using SEM, TEM, and EDS mapping, as shown in Figure 2.

Figure 2a displays the SEM image of carbon-coated VN nanosheets (VN@CNS), revealing a uniform spherical morphology with an average particle size of approximately 50 nm. This homogeneous size distribution facilitates consistent electrochemical reaction kinetics.

After the incorporation of carbon nanofibers (CNFs), the SEM image in Figure 2b shows that the VN@CNS nanoparticles are tightly anchored onto interconnected carbon nanofibers with diameters ranging from 40 to 200 nm. This three-dimensional conductive network not only provides efficient pathways for rapid electron transport but also creates abundant ion diffusion channels, which are crucial for enhancing rate capability and electrochemical accessibility.

At higher magnifications (Figure 2c), the hierarchical configuration of the VN@CNS/CNF composite is clearly observed. Numerous carbon-modified VN nanoparticles with an average diameter of approximately 5–10 nm are uniformly dispersed and firmly anchored on the surface of nitrogen-doped carbon nanofibers. This refined architecture ensures intimate interfacial contact between the active VN and the conductive carbon matrix, effectively shortening the ion/electron transport paths.

TEM analysis provides further insights into the structural configuration. As shown in Figure 2d, the carbon nanofiber exhibits a uniform tubular morphology, and numerous VN@CNS nanoparticles are tightly embedded along the fiber surface (Figure 2e).

The high-resolution TEM (HRTEM) image (Figure 2f) reveals distinct lattice fringes with a d-spacing of 0.21 nm, corresponding to the (200) crystal plane of VN, confirming the successful formation of crystalline VN nanoparticles within the carbon framework. The intimate interfacial contact between VN and the carbon matrix is expected to significantly shorten the ion/electron transport distance and enhance electrochemical reversibility.

Elemental mapping results (Figure 2g) further verify the uniform distribution of V, N, and C elements throughout the nanofiber structure, demonstrating the homogeneous integration of VN nanoparticles and nitrogen-doped carbon layers. Such a synergistic configuration not only prevents VN nanoparticle agglomeration but also provides abundant electroactive sites and robust mechanical stability.

#### 3.1.2. XRD and Raman Spectroscopy Analysis

**Figure 3:**
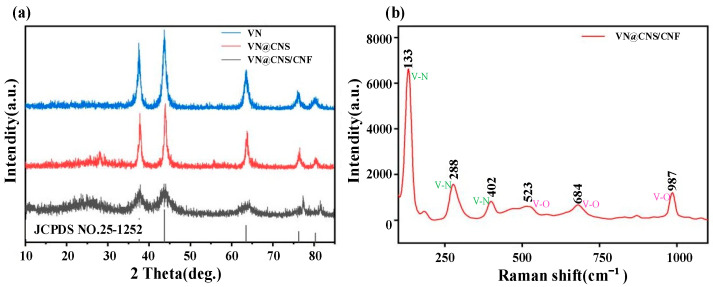
(**a**) XRD patterns of VN@CNS and VN@CNS/CNF; (**b**) Raman spectra of VN@CNS/CNF.

The crystalline structures and phase compositions of VN, VN@CNS, and VN@CNS/CNF composites were analyzed by X-ray diffraction (XRD) and Raman spectroscopy, as shown in Figure 3.

In the XRD patterns (Figure 3a), all samples exhibit characteristic diffraction peaks at approximately 37.7°, 43.7°, 63.6°, 76.4°, and 80.3°, corresponding to the (111), (200), (220), (311), and (222) planes of cubic VN (JCPDS No. 25-1252), confirming the successful synthesis of crystalline VN. Compared with pure VN, the VN@CNS and VN@CNS/CNF composites show slightly broadened diffraction peaks and reduced intensity, which can be attributed to the carbon coating layer and the nanoscale dispersion of VN particles within the conductive carbon matrix. This structural modulation not only enhances the interfacial contact between VN and the carbon framework but also optimizes the electron transport pathways, thereby improving the overall electrical conductivity and electrochemical reactivity. In addition, the broad peak around 25° is attributed to the (002) plane of graphitic carbon, indicating partial graphitization within the carbon matrix. The absence of any impurity peaks further confirms the high phase purity and structural integrity of the synthesized composite.

To further evaluate the structural features of the VN@CNS/CNF sample, Raman spectroscopy was carried out (Figure 3b). The characteristic peaks located at 133 cm^−1^, 288 cm^−1^, and 402 cm^−1^ are assigned to V–N vibrations, while those at 523 cm^−1^, 684 cm^−1^, and 987 cm^−1^ correspond to V–O stretching modes. The coexistence of both V–N and V–O bonds indicates the formation of a heterointerface between VN nanoparticles and the carbon framework, which enhances structural robustness and provides active sites for Zn^2+^ storage.

#### 3.1.3. Nitrogen Adsorption–Desorption and Thermogravimetric Analysis

**Figure 4:**
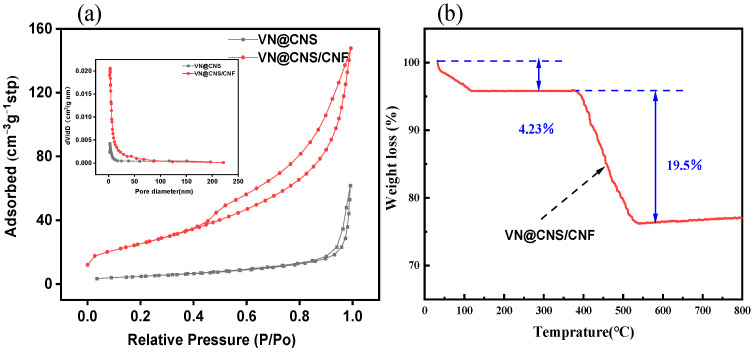
(**a**) N_2_ adsorption–desorption isotherms and corresponding pore size distribution of VN@CNS and VN@CNS/CNF; (**b**) TGA curve of VN@CNS/CNF.

The porosity and composition of the synthesized samples were investigated using nitrogen adsorption–desorption and thermogravimetric analysis (TGA). As shown in Figure 4a, the VN@CNS/CNF composite exhibits a typical type IV isotherm with an H_3_ hysteresis loop, suggesting a well-developed mesoporous structure. The Brunauer–Emmett–Teller (BET) specific surface area is 87.81 m^2^g^−1^, which is significantly larger than that of VN@CNS (15.94 m^2^g^−1^). Such a hierarchical porous structure promotes electrolyte infiltration and accelerates ion transport, contributing to enhanced electrochemical performance.

The TGA curve (Figure 4b) reveals two major weight loss stages. The initial loss of approximately 4.23% from 50 °C to 140 °C corresponds to the removal of adsorbed and crystalline water. A subsequent weight loss of about 19.5% in the range of 360–800 °C is attributed to the oxidative decomposition of the carbon component. Based on this analysis, the VN content in the VN@CNS/CNF composite is estimated to be around 76.3 wt%, which is in good agreement with the XRD results, confirming that VN is the dominant crystalline phase within the composite, the detail explanation can be seen in the Supplementary Material File.

These findings collectively demonstrate that the VN@CNS/CNF composite features high VN loading, a large specific surface area, and abundant mesopores, providing numerous electrochemically active sites and efficient ion diffusion channels for reversible Zn^2+^ storage.

#### 3.1.4. X-Ray Photoelectron Spectroscopy (XPS) Analysis

**Figure 5:**
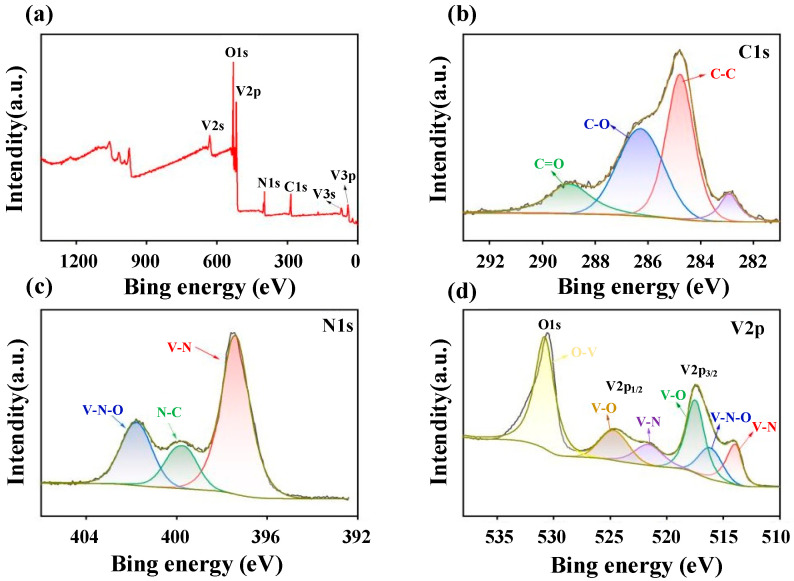
(**a**) Survey X-ray photoelectron spectroscopy (XPS) spectrum of VN@CNS/CNF composite; (**b**) C 1s spectrum; (**c**) N 1s spectrum; (**d**) O 1s and V 2p spectra.

To further investigate the chemical composition and valence states of the VN@CNS/CNF composite, X-ray photoelectron spectroscopy (XPS) was performed. As shown in Figure 5a, the full XPS survey spectrum clearly reveals the presence of V, N, O, and C elements, confirming their successful incorporation into the composite structure. It is noteworthy that metal nitrides are prone to surface oxidation upon exposure to air, leading to the formation of V–O species on the surface [29,30]. Such surface oxidation is confined to a few nanometers and does not alter the VN crystal structure, as supported by XRD and HRTEM analyses (Figure 2f), which show well-defined VN (200) lattice fringes without any detectable oxide phase. The presence of this ultrathin V–O_x_ layer can actually be beneficial, as it promotes interfacial redox reactions and facilitates charge transfer by improving electrolyte wettability and providing additional surface-active sites [31].

The high-resolution C 1s spectrum (Figure 5b) exhibits three distinct peaks centered at 284.8, 286.3, and 289.0 eV, corresponding to C–C, C–O, and C=O bonds, respectively [32], consistent with a carbonaceous shell containing residual oxygenated functional groups. These surface functionalities enhance the interfacial wettability and electronic conductivity of the carbon framework. The N 1s spectrum (Figure 5c) shows three characteristic peaks at 401.8, 399.9, and 397.4 eV, assigned to V–N–O, N–C, and V–N bonds, respectively, indicating that nitrogen atoms are not only bonded to vanadium but also doped into the carbon network, thereby contributing to the modulation of the electronic environment. The V 2p spectrum (Figure 5d) further confirms the coexistence of multiple vanadium valence states: the V 2p_3/2_ peaks at 517.6, 516.5, and 514.1 eV correspond to V–O, V–N–O, and V–N bonds, respectively, while the V 2p_1/2_ peaks at 524.4 and 521.8 eV are assigned to V–O and V–N bonds [33].

These observations are consistent with the Raman analysis (Figure 3b) and collectively demonstrate the coexistence of V–N, V–O, and C–N bonds within the VN@CNS/CNF composite. The in situ-formed conductive carbon network not only ensures efficient and stable electron transport but also enhances the mechanical robustness of the electrode through these strong chemical linkages. This synergistic structural design effectively mitigates active material pulverization and aggregation during cycling, thereby improving the overall structural integrity and long-term cycling stability of the electrode.

### 3.2. Electrochemical Performance of VN@CNS/CNF Electrodes

#### 3.2.1. Cyclic Voltammetry and Galvanostatic Charge–Discharge Performance

**Figure 6:**
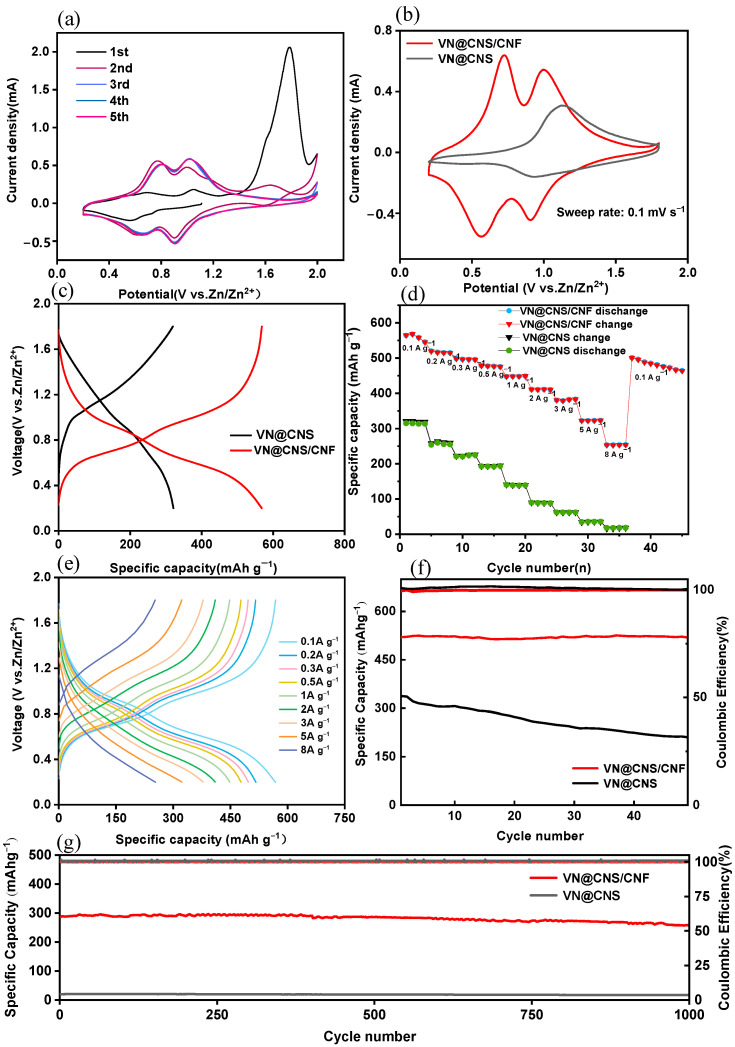
Electrochemical performance of VN@CNS and VN@CNS/CNF electrodes. (**a**) CV curves of VN@CNS/CNF at first five cycles (0.1 mV s^−1^); (**b**) comparison of CV curves at 5th cycle between VN@CNS and VN@CNS/CNF at scan rate of 0.1 mV s^−1^; (**c**) galvanostatic charge–discharge (GCD) curves at current density of 0.1 A g^−1^; (**d**) rate performance of VN@CNS and VN@CNS/CNF at different current densities; (**e**) corresponding GCD curves of VN@CNS/CNF under different current densities; (**f**) cycling performance and coulombic efficiency at current density of 0.1 A g^−1^; (**g**) long-term cycling stability and coulombic efficiency at high current density of 8 A g^−1^.

Figure 6 comprehensively demonstrates the electrochemical performance of the VN@CNS/CNF composite electrode compared with the VN@CNS counterpart.

In Figure 6a, the cyclic voltammetry (CV) curves of VN@CNS/CNF over the first five consecutive cycles display highly consistent redox peak positions and current responses, indicating excellent electrochemical reversibility and structural stability during repeated Zn^2+^ insertion/extraction. Three anodic peaks can be clearly observed in the first cycle, located at approximately 0.9 V, 1.3 V, and 1.7–1.9 V. The first two peaks correspond to the multistep oxidation of V^2+^/V^3+^ and V^3+^/V^4+^ redox couples during Zn^2+^ extraction, while the additional high-potential peak (1.6–2.0 V) originates from the further oxidation of intermediate vanadium species (e.g., V^3+^/V^4+^) into higher-valence V^5+^ states. This irreversible activation process stabilizes after the initial cycle, resulting in nearly overlapping CV profiles in subsequent scans, which confirms enhanced reversibility and structural integrity [34].

In Figure 6b, the CV curves of the fifth cycle are compared for VN@CNS/CNF and VN@CNS electrodes. Both electrodes exhibit similar redox characteristics; however, VN@CNS/CNF shows two dominant pairs of cathodic and anodic peaks at 0.725/1.00 V and 0.566/0.905 V, respectively, corresponding to the multistep Zn^2+^ insertion/extraction in VN. Compared with VN@CNS, the VN@CNS/CNF electrode presents sharper and more symmetric peaks with higher current densities, indicating faster redox kinetics and lower polarization [35]. These advantages arise from the conductive carbon nanofiber framework and the intimate VN–CNS interface, which together promote efficient electron transport and rapid ion diffusion.

The GCD curves in Figure 6c further confirm these results. VN@CNS/CNF exhibits a smaller voltage hysteresis and higher discharge capacity compared with VN@CNS at the same current density (0.1 A g^−1^), suggesting enhanced reversibility and reduced internal resistance.

The rate capability, as displayed in Figure 6d, demonstrates that VN@CNS/CNF delivers a high specific capacity of 564 mAh g^−1^ at 0.1 A g^−1^ and maintains 280 mAh g^−1^ even at 8 A g^−1^, outperforming VN@CNS across all current densities. This superior rate performance is attributed to the hierarchical conductive architecture, where the interconnected carbon nanofibers accelerate electron transport and the carbon-coated VN nanoparticles facilitate rapid Zn^2+^ diffusion and suppress polarization.

In contrast, the GCD curves at different current densities (Figure 6e) emphasize the reversibility of the electrochemical process rather than rate capability. The nearly overlapping charge–discharge plateaus indicate stable redox behavior, low voltage hysteresis, and robust electrode integrity during rapid cycling. Thus, Figure 6d highlights the fast charge adaptability of VN@CNS/CNF.

The cycling test at 0.1 A g^−1^ (Figure 6f) shows that VN@CNS/CNF maintains 99% of its initial capacity after 50 cycles with nearly 100% coulombic efficiency, far exceeding the 62.31% retention of VN@CNS, confirming its outstanding reversibility and minimal side reactions. This outstanding stability is attributed to the synergistic effect of the carbon coating and the 3D conductive carbon network, which reinforce structural integrity and mitigate vanadium dissolution. Notably, when the current density returns to 0.1 A g^−1^, the specific capacity recovers to 530 mAh g^−1^, demonstrating excellent reversibility and structural robustness even under high-rate cycling conditions.

Furthermore, the long-term cycling performance at 8 A g^−1^(Figure 6g) demonstrates remarkable durability, with a stable capacity of approximately 280 mAh g^−1^ retained after 1000 continuous cycles and coulombic efficiency remaining above 99%. In contrast, VN@CNS delivers merely 18 mAh g^−1^ under the same conditions. These results highlight the superior structural robustness and charge transfer stability of the VN@CNS/CNF electrode under high-rate conditions.

Overall, the VN@CNS/CNF electrode exhibits significantly improved electrochemical kinetics, rate capability, and cycling durability compared to VN@CNS. This improvement is attributed to the synergistic cascade architecture, where the conductive carbon nanofiber network ensures rapid electron transport, while the carbon-coated VN nanoparticles provide abundant active sites and suppress volume fluctuations during cycling.

#### 3.2.2. Electrochemical Kinetics Analysis

**Figure 7:**
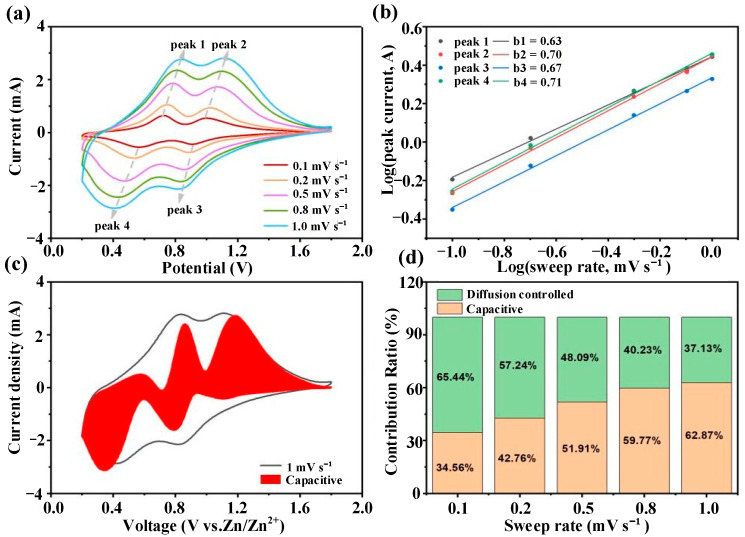
(**a**) CV curves at different scan rates; (**b**) the relationship between redox peaks and scan rates was examined by plotting log(i) versus log(v) based on the CV data; (**c**) the capacitance separation curve at a scan rate of 1 mV s^−1^; (**d**) the contribution of capacitive behavior to the overall capacity was analyzed at various scan rates.

To elucidate the electrochemical response characteristics of the VN@CNS/CNF electrode, cyclic voltammetry (CV) measurements were performed at scan rates ranging from 0.1 to 1.0 mV s^−1^. As illustrated in Figure 7a, compared with the VN@CNS electrode, the VN@CNS/CNF composite exhibits significantly enhanced electrochemical reversibility, cycling stability, and rate capability. With increasing scan rate, the redox peaks gradually shift—oxidation peaks move toward higher potentials while reduction peaks shift to lower potentials—indicating increased polarization associated with intrinsic charge transfer kinetics.

The capacity contribution behavior can be analyzed using the kinetics equation relating the peak current (*i*) to the scan rate (*v*) [36]:

(1) $$i=av^{b}$$

where *a* and *b* are adjustable parameters, *v* is the scan rate (V s^−1^), *i* is the current (A), and *v* is the scan rate. A *b* value of 0.5 indicates a diffusion-controlled process, while a *b* value close to 1.0 corresponds to a surface-controlled capacitive process [37,38].

As shown in Figure 7b, the *b*-values of the four redox peaks are 0.63, 0.70, 0.67, and 0.71, respectively, confirming the dominant contribution of pseudocapacitive processes to the total capacity.

To quantitatively differentiate between capacitive and diffusion-controlled contributions, the total current (*i*) can be fitted as [39,40]

*i* = *k*_1_*v*^1/2^ + *k*_2_*v*, (2)

where *k*_1_*v*^1/2^ and *k*_2_*v* correspond to the diffusion-controlled and capacitive-controlled processes, respectively.

At a scan rate of 0.5 mV s^−1^, the capacitive contribution accounts for 51.91% of the total charge storage (Figure 7c,d). With increasing scan rate from 0.1 to 1.0 mV s^−1^, the capacitive contribution ratio rises from 34.56% to 62.87% (Figure 7c), indicating a progressively enhanced pseudocapacitive behavior.

These findings suggest that as the scan rate increases, the energy storage mechanism of VN@CNS/CNF transitions from a diffusion-controlled process to a surface-dominated pseudocapacitive behavior. This transition facilitates faster charge transport and promotes rapid, reversible Zn^2+^ insertion/extraction at the electrode–electrolyte interface, thereby enabling the composite to achieve outstanding rate capability and cycling performance.

#### 3.2.3. Electrochemical Impedance Spectroscopy (EIS)

**Figure 8:**
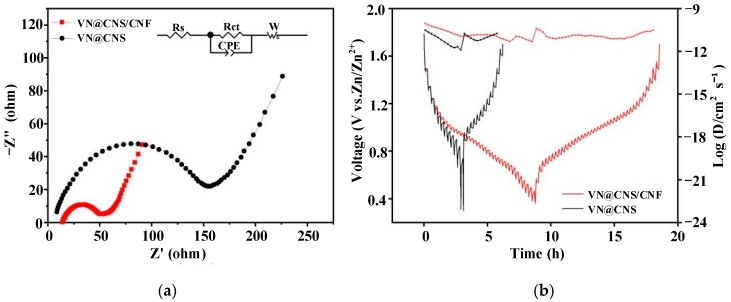
(**a**) EIS spectra fitted using the equivalent circuit model; (**b**) GITT curves of VN@CNS/CNF and VN@CNS electrodes.

Electrochemical impedance spectroscopy (EIS) was employed to investigate the charge transfer characteristics of the electrode materials [41]. As illustrated in Figure 8a, the impedance spectra of VN@CNS/CNF and VN@CNS electrodes exhibit distinct semicircles in the high-frequency region, which correspond to the charge transfer resistance (Rct). The Rct value of the VN@CNS/CNF electrode (52.1 Ω) is markedly lower than that of VN@CNS (154.3 Ω), indicating significantly enhanced charge transfer kinetics. This reduction in resistance can be attributed to the in situ interconnected VN nanorods within the conductive carbon nanofiber network, which effectively facilitates electron transport and improves overall conductivity. Moreover, the Nyquist plot of VN@CNS/CNF displays a steeper linear slope in the low-frequency region, suggesting improved Zn^2+^ diffusion behavior within the electrode structure. These results collectively demonstrate that the VN@CNS/CNF composite exhibits superior interfacial charge transport and ion diffusion performance.

To further evaluate the Zn^2+^ transport dynamics, galvanostatic intermittent titration technique (GITT) measurements were performed, as shown in Figure 8b. The diffusion coefficients of Zn^2+^ ions in the VN@CNS/CNF electrode are significantly higher than those in VN@CNS during both the charging and discharging processes, confirming faster ion transport dynamics. This improvement is primarily attributed to the nitrogen-doped carbon coating on the VN surface, which modifies the local electronic environment and promotes Zn^2+^ mobility. In addition, the strong interfacial bonding between VN nanoparticles and carbon nanofibers forms a multidimensional conductive network. Such architecture not only increases the number of electroactive sites but also minimizes interfacial resistance and enhances the charge transfer kinetics, thereby facilitating rapid and reversible Zn^2+^ insertion/extraction.

Overall, the integration of VN nanorods, nitrogen-doped carbon coating, and carbon nanofibers constructs a hierarchical VN@CNS/CNF composite that exhibits a balanced combination of fast charge transfer, efficient ion diffusion, and stable interface chemistry. The thin surface V–O shell—confirmed by Raman and XPS analysis—plays a beneficial role by promoting redox pseudocapacitance without introducing substantial resistance, consistent with the EIS and GITT findings. These results collectively demonstrate that the VN@CNS/CNF composite achieves superior electrochemical kinetics through the synergistic coupling of conductive networks and controlled surface oxidation.

## 4. Conclusions

In this study, a tandem-like vanadium nitride@carbon nanosheet/carbon nanofiber (VN@CNS/CNF) composite was successfully synthesized via a facile hydrothermal self-assembly combined with high-temperature calcination, based on a cascade structure-guided electron transport optimization strategy. The unique tandem architecture effectively optimizes the electron and ion transport pathways, thereby enhancing electrical conductivity and charge transfer kinetics. Meanwhile, the conformal carbon coating serves to mitigate the volume expansion of VN during repeated charge–discharge cycles, improving the structural integrity of the electrode. Electrochemical evaluations reveal that the VN@CNS/CNF electrode delivers a high specific capacity of 564 mAh g^−1^ at 0.1 A g^−1^, with a remarkable capacity retention of 99% after 50 cycles. Even at a high current density of 8 A g^−1^, the electrode still maintains a capacity of 280 mAh g^−1^. These outstanding electrochemical performances—including high capacity, excellent rate capability, and superior cycling stability—demonstrate that the VN@CNS/CNF composite is a promising electrode material, providing valuable insights for the development of advanced aqueous zinc-ion batteries.

## Data Availability

All the datasets used in this manuscript are publicly available datasets already in the public domain.

## Supplementary Materials

The following supporting information can be downloaded at: https://www.mdpi.com/article/10.3390/mi16111265/s1.
