# Supplementary material for: Carbon NanoFiber-Integrated VN@CNS Multilevel Architectures for High-Performance Zinc-Ion Batteries
Source: Micromachines (Basel). 2025 Nov 10;16(11):1265. doi: 10.3390/mi16111265 (PMC12654288; doi:10.3390/mi16111265)
Supplement: Supplementary file 1 [file micromachines-16-01265-s001.zip › micromachines-3937380-supplementary.pdf]

To test whether the measured VN loading is chemically plausible given the synthesis stoichiometry, a simple mass-balance estimate using the reagent amounts used in the reported synthesis (1.5 mmol NH_4VO_3 and 4.5 mmol melamine) is performed:

Molar mass: VN = M(V) + M(N) ≈ 50.94 + 14.01 = 64.95 g mol^{-1}.

If all V from 1.5 mmol NH_4VO_3 is converted to VN: mass(VN) = 1.5×10^{-3} mol × 64.95 g mol^{-1} = 0.0974 g.

Initial carbon available from 4.5 mmol melamine ($C_3H_6N_6$; M = 126.12 g mol^{-1}): carbon content per melamine molecule = 3×12.011 = 36.033 g mol^{-1}. Thus initial carbon mass = 4.5×10^{-3} mol × 36.033 g mol^{-1} = 0.1625 g (this is the maximum carbon mass before pyrolysis losses).

If a fraction y of the initial melamine carbon remains as solid carbon after pyrolysis/carbonization, the expected VN mass fraction is:

$$\text{VN wt\%} = \frac{m_{\text{VN}}}{m_{\text{VN}} + y \cdot m_{\text{C,initial}}} \quad \text{(S1)}$$

Solving for y that yields the experimentally measured VN wt% (0.7627):

$$y = \frac{m_{\text{VN}}\left(\frac{1}{0.7627} - 1\right)}{m_{\text{C,initial}}} \approx 0.186 (\text{or } 18.6\%) \quad \text{(S2)}$$

An effective carbon yield of ~18 – 20% from melamine under our carbonization conditions is reasonable (melamine decomposes extensively, and only a fraction remains as solid carbon), so the stoichiometric estimate is consistent with the experimental VN fraction.

To quantitatively verify the VN loading fraction reported here (76.27 wt% for VN@CNS/CNF), a stoichiometric estimation was performed based on precursor composition and compared with the values obtained from TGA and XRD Rietveld refinement, as summarized in Table 1.

Table S1. Stoichiometric estimate and cross-validation of VN mass fraction in VN@CNS/CNF.

Item	Value (Numeric)	Notes / Assumption
NH_4VO_3 amount used	1.5 mmol	Synthesis precursor (reported)
molar mass VN (V+N)	64.95 g mol^{-1}	M(V)=50.94, M(N)=14.01
Theoretical VN mass (if all V → VN)	0.09743 g	1.50 mmol × 64.95 g mol^{-1}
Melamine amount used	4.50 mmol	Synthesis precursor (reported)
C atoms per melamine molecule	3 atoms per molecule	melamine = $C_3H_6N_6$
Carbon mass per mol melamine	36.033 g mol^{-1}	3 × 12.011 g mol^{-1}
Initial carbon mass from melamine	0.16215 g	4.50 mmol × 36.033 g mol^{-1}
Assumed carbon retention (solid yield) after pyrolysis (y)	18.7%	value required to match measured VN wt%; consistent with typical melamine carbon yield under these conditions
Calculated VN wt% (stoichiometric balance)	76.27 wt%	= m_VN / (m_VN + y m_C_initial) → 0.09743 / (0.09743 + 0.187×0.16215)
TGA-derived VN wt% (experimental)	76.27 wt%	Figure 4b: mass losses due to water (~4.23%) and carbon combustion (~19.5%)
XRD (Rietveld) derived VN wt%	75.9 wt%	quantitative phase analysis (Rietveld refinement); Rwp ≈ 7.8%, $\chi^2 \approx 1.9$
